# The Role of Spatial Aortic Arch Architecture in Type B Aortic Dissection

**DOI:** 10.3390/jcm12185963

**Published:** 2023-09-14

**Authors:** Joscha Mulorz, Franziska Garcon, Amir Arnautovic, Casper De Somer, Artis Knapsis, Hug Aubin, Felix Fleissner, Julian-Dario Rembe, Malwina Vockel, Alexander Oberhuber, Artur Lichtenberg, Hubert Schelzig, Markus Udo Wagenhäuser

**Affiliations:** 1 Clinic for Vascular and Endovascular Surgery, Medical Faculty and University Hospital Düsseldorf, Heinrich-Heine-University, 40225 Düsseldorf, Germany; 2 Institute for Biomedical Engineering and Technology, Ghent University, 9000 Ghent, Belgium; 3 Department of Cardiac Surgery, Medical Faculty and University Hospital Duesseldorf, Heinrich-Heine-University, 40225 Düsseldorf, Germany; 4 CURE3D Laboratory, Medical Faculty and University Hospital Düsseldorf, Heinrich-Heine-University, 40225 Düsseldorf, Germany; 5 Department of Vascular and Endovascular Surgery, University Hospital Muenster, 48149 Muenster, Germany; 6 Cardiovascular Research Institute Düsseldorf (CARID), Heinrich-Heine-University, 40225 Düsseldorf, Germany; 7 Department of Vascular- and Endovascular Surgery, University Hospital Düsseldorf, Moorenstraße 5, 40225 Düsseldorf, Germany

**Keywords:** type B aortic dissection, aortic arch, morphology, supra-aortic branches

## Abstract

Objective: The incidence of type B aortic dissection (TBAD) is increasing worldwide; however, the underlying pathomechanisms are not conclusively understood. This study explores the geometric architecture of the aortic arch and supra-aortic branches in TBAD patients as opposed to non-TBAD patients. Methods: Patient characteristics were retrieved from archived medical records. Computer-assisted tomography (CAT) scans of patients with TBAD and carotid stenosis (CS) from two high-volume centers were analyzed. Various aortic arch parameters and take-off angles of the supra-aortic branches of TBAD patients were measured following centerline normalization in comparison CS patients. A compression index (C-index) was calculated from the para-sagittal, and a torsion index (T-index) was calculated from the para-coronal take-off angles of the supra-aortic branches to analyze aortic arch tortuosity. Results: A total of 199 CAT scans were analyzed, namely, 85 in the TBAD group and 114 in the CS group. The average age was 61.5 ± 13.1 years among the TBAD patients and 71 ± 9.3 years among the CS patients. We found a significantly higher proportion of type III aortic arch configurations in TBAD patients compared with CS patients. Further, the aortic arch angle was steeper in the TBAD group. In the para-sagittal plane, the left subclavian artery (LSA) take-off angle was less steep in TBAD patients. In the para-coronal plane, the left carotid artery (LCA) had a less steep take-off angle, while the LSA had a more obtuse take-off angle in the TBAD group when compared with the CS group. In addition, the inter-vessel distance was increased in TBAD patients. Finally, the T-index was increased, suggesting a significant torsion resulting from the deviating take-off angles of the supra-aortic branches supplying the left half of the body as opposed to the innominate artery (IA) in TBAD patients. Conclusions: Our results suggest several aortic arch-specific geometric configurations in patients suffering from TBAD that significantly differ from those in CS patients. Further functional studies are needed to verify the pathogenetic relevance of our results and their disease-specific causality. Although our data are not mechanistically explorative, they may serve as a basis for identifying future patients with aortic arch morphology at higher risk for TBAD development and who may benefit from more stringent adjustment of risk factors as a primary prevention concept.

## 1. Introduction

Acute aortic dissection (AAD), first described by Frank Nicholls following the autopsy of King George II, is a severe cardiovascular disease accounting for 30-day mortality rates of up to 57% [1]. It is one of the major diagnoses of acute aortic syndrome (AAS), which also encompasses intramural hematoma (IMH) and penetrating aortic ulcers (PAU). Although the mortality rate is significantly lower among patients suffering from type B aortic dissection (TBAD) when compared with type A aortic dissection (TAAD), which involves an ascending aorta, TBAD remains a significant health burden for patients and health care systems due to long-term post-incidence surveillance and potential complications such as dissecting aneurysm formation [2].

Consequently, there is high interest in preventing the initial intimo-medial entry tear, which allows blood to flow between the layers, causing de-lamination while propagating within the aortic wall [3]. Risk factors for developing TBAD include the male sex, advanced age, pregnancy, hypertension, dyslipidemia, genetic predisposition, and aortic enlargement among others [4,5]. Given the demographic shift in most industrialized countries with an increasing prevalence of cardiovascular risk factors, developing primary prevention concepts to identify patients at risk of developing severe cardiovascular diseases such as TBAD is eminently relevant [6].

In this context, aortic arch configuration is becoming increasingly important since anatomic variants of the aorta have been linked to TBAD development [7]. To this end, a so-called type III arch configuration, or gothic arch, with the top of the arch located at the distal end of the supra-aortic branches, has been reported to be associated with TBAD manifestation [8]. Although the link between aortic arch morphology and TBAD development seems conclusive, the configuration and position of the supra-aortic vessels in relation to the aortic arch have yet to be investigated.

Therefore, the present study evaluates various aortic arch geometry parameters in TBAD and non-TBAD patients assessed by computer-assisted tomography (CAT) scans from two German high-volume centers.

## 2. Material and Methods 

### 2.1. Data Collection 

**Figure 1:**
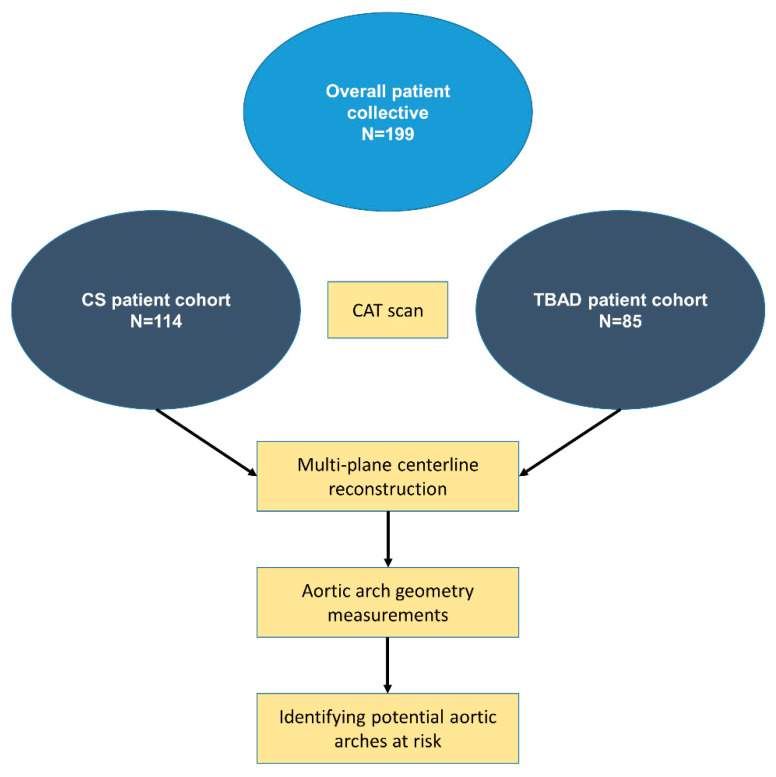
Schematic workflow diagram. The overall patient collective consisted of 199 patients. A total of 114 computer-assisted tomography (CAT) scans from patients with carotid stenosis (CS) and 85 with type B aortic dissections (TBAD) were analyzed. Following multi-plane centerline reconstruction, various geometric parameters of the aortic arch were analyzed to identify potential aortic arches at risk, by comparing the two groups.

In this retrospective clinical study, we included patients diagnosed with TBAD with at least one CAT scan at the time of admission at the University Hospital Düsseldorf, Germany, and the University Hospital Münster, Germany, between 1 January 2006 and 31 June 2019, and 1 February 2013 and 31 March 2020, respectively. For the control group, we included patients diagnosed with carotid stenosis (CS) with one available CAT scan prior to treatment. The relevant data were retrieved from archived medical records at the time of diagnosis and CAT scans for subsequent analysis (Figure 1).

### 2.2. CAT Scan Analysis 

The CAT scans of TBAD and CS patients were exported from the Picture Archiving and Communication System (PACS) to the open-source analysis tool Horos (version 4.0.0 RC5; https://horosproject.org/).

**Figure 2:**
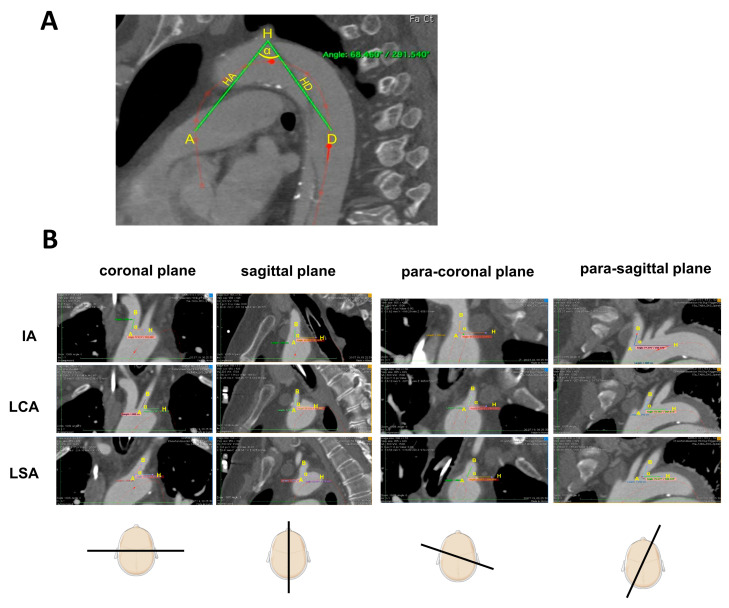
Aortic arch angles and supra-aortic branch offspring angles. (**A**) The aortic arch angle was measured in a sagittal plane. The angle α was defined by three points. Points A and D were placed at the level of the pulmonary bifurcation onto the centerline of the ascending (A) and descending (D) aorta, respectively. Point H was placed on the highest possible point of the aortic arch in the sagittal plane. (**B**) Supra-aortic branch offspring angles measurements. To investigate the geometric configuration of the supra-aortic branches in relation to the aortic arch, the offspring angle of these branches was measured in the (para-)sagittal and (para-)coronal planes following multi-plane centerline reconstruction. Each angle was defined by a line between two well-defined points against the horizontal. The procedure was identical in the coronal and sagittal planes. For the angle (α), point A was set in the center of the branch ostium. Then, point B was placed in the middle of the supra-aortic branch at an exact distance of 10 mm from point A. The line defined by this was measured against the horizontal for angle determination. IA: innominate artery. LCA: left carotid artery. LSA: left subclavian artery. The graphic was in part created with Biorender (BioRender, Toronto, Canada).

Various morphologic parameters of the aortic arch were assessed for each patient. Then, the aortic arch configuration was classified based on a commonly used three-category system, which is defined by the vertical distance from the origin of the innominate artery (IA) to the top of the aortic arch. Based on this measurement, aortic arches were categorized as follows: (1) type 1 arch—distance < 1 diameter of the left common carotid artery (CCA); (2) type II arch—distance between 1 and 2 CCA diameters; and (3) type III arch—distance > 2 CCA diameters [9]. The aortic arch angle was measured in the sagittal plane at three given points. To elaborate, two points were placed at the level of the pulmonary bifurcation on the centerline of the ascending and descending aortas. The third point was placed at the most apical point in the aortic arch in the sagittal plane (Figure 2A).

The spatial geometry of the supra-aortic branches was determined according to previously established methods [10]. In short, the take-off angles of the supra-aortic branches were measured in coronal and sagittal views with or without centerline normalization against the horizontal line (Figure 2B). The distance between the two supra-aortic branches was measured following centerline reconstruction and was defined between the proximal edges of the ostium of each of the two side branches.

A compression index (C-index) and torsion index (T-index) were calculated to evaluate the potential compression and/or torsion of the aortic arch due to the spatial configuration of the take-off angles of the supra-aortic branches. This was calculated separately for the sagittal and coronal planes using the para-sagittal and para-coronal take-off angles for all supra-aortic branches, respectively. The rationale of the indices is to quantify the deviation of the take-off angles of the left carotid artery (LCA) and/or the left subclavian artery (LSA) with respect to the IA. In the sagittal view, this indicates a sprain or compression, whereas in the coronal view, it indicates torsion. The indices were calculated as follows:

$$\begin{array}{ll} \mathrm{C-index}\;1: & \left|IA_{para-sag}-LCA_{para-sag}\right|\\ \mathrm{C-index}\;2: & \left|IA_{para-sag}-LSA_{para-sag}\right|\\ \mathrm{C-index}\;\mathrm{total}: & C-index\;1+C-index\;2\\ \mathrm{T-index}\;1: & \left|IA_{para-cor}-LCA_{para-cor}\right|\\ \mathrm{T-index}\;2: & \left|IA_{para-cor}-LSA_{para-cor}\right|\\ \mathrm{T-index}\;\mathrm{total}: & T-index\;1+T-index\;2 \end{array}$$

### 2.3. Statistical Analysis 

Categorical variables are presented as relative frequencies with percentages, while continuous variables are shown as mean and standard deviation (SD). The Shapiro–Wilk test was used for the assessment of normal distribution. The Chi-square test was applied for categorical variables. Finally, in cases where *n* < 5, Fisher’s exact test was applied. After testing for normality, normally distributed variables are presented as mean and SD and compared using the Student’s *t*-test, while non-normally distributed variables are presented as the median and interquartile range and compared using the Mann–Whitney *U* test. Further, estimation plots are presented as the mean with 95% confidence intervals (CI). A *p*-value of <0.05 was considered statistically significant. Statistical tests were performed using GraphPad Prism (GraphPad Software, Inc., version 10.0.1, La Jolla, CA, USA).

### 2.4. Ethical Approval

The study was approved by the local ethics committees at Heinrich Heine University Düsseldorf, Germany, and Westphalian Wilhelm University Münster, Germany (approval IDs 2019-635/2019-635_2 and 2019-764-b-S). It followed all applicable standards for good scientific practice and the Declaration of Helsinki.

## 3. Results 

### 3.1. Patient Characteristics

We analyzed the CAT scans of 199 patients, 114 of whom were scanned for CS and 85 for TBAD prior to any kind of surgical intervention. The mean slice thickness was 2.7 ± 2.2 mm for the TBAD group and 1 ± 0.7 mm for the CS group.

Patients in the CS group were older, with more than half of the patients’ ages ranging from 65 to 79 years. There were no significant differences in gender distribution, weight, or body mass index. In both groups, a high percentage of patients were on anti-hypertensive medication, which did not differ significantly among both groups except for AT antagonists that were prescribed more frequently in the CS group. Interestingly, more patients were diagnosed with hypertension in the CS group when compared with the TBAD patients. Furthermore, in the CS group, there were more diabetics and patients with a history of smoking, indicating an elevated cardiovascular risk profile in this cohort (Table 1).

### 3.2. Anatomical Parameters of the Aortic Arch and Supra-Aortic Branches

Various anatomic and morphologic aortic arch parameters were analyzed to identify disease-specific differences.

**Figure 3:**
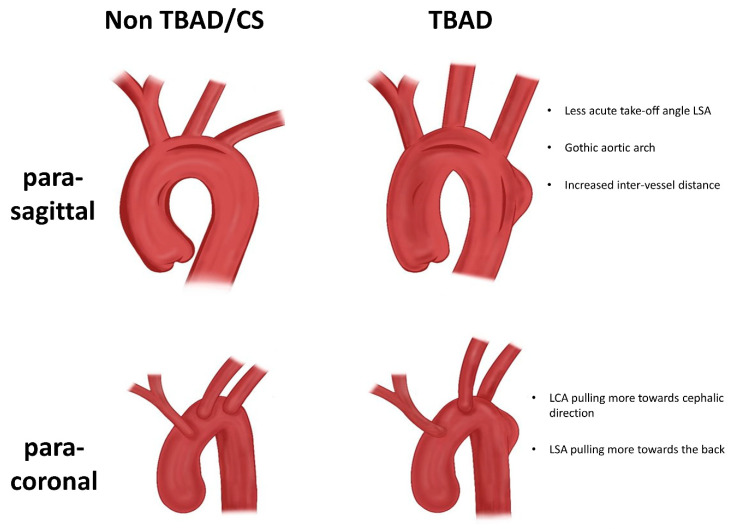
Schematic overview of the spatial geometric differences in the aortic arch configuration. In the para-sagittal plane, the left subclavian artery (LSA) revealed a less acute take-off angle in the type B aortic dissection (TBAD) group. The aortic arch angle was steeper in the TBAD group than in the carotid artery stenosis (CS) group; in other words, a gothic aortic arch configuration was more common in TBAD group. The distance between the proximal edge of the ostium between each supra-aortic branch was increased in the TBAD vs. the CS group. In the para-coronal plane, the left carotid artery (LCA) orients more towards the cephalic direction, and the LSA orients more to the back. No differences in the spatial configuration were observed for the innominate artery (IA). Own drawing created using Procreate (Savage Interactive, Hobart, Australia, version 5.3.5.).

We found characteristic differences in the anatomical configuration of the aortic arch and the supra-aortic vessels in the TBAD group compared with the CS group. All relevant differences in aortic arch morphology are presented in Figure 3.

Specifically, a type III arch configuration occurred more frequently in the TBAD group, while type II was more frequent in the CS group. Conclusively, a gothic aortic arch configuration with a characteristic steeper angle between ascending and descending aortas was more common in TBAD patients (Table 2).

In addition to the morphological characteristics of the aortic arch, the spatial outflow configuration of the supra-aortic branches was analyzed. It is noteworthy that the take-off angles did not differ significantly for the IA in either the (para-)sagittal or the (para-)coronal plane between TBAD and CS patients. Thus, we found only differences in the spatial configuration of the supra-aortic branches supplying the left half of the body.

On the one hand, for the LCA, which was measured against the horizontal, there was a less acute take-off angle in the (para-)coronal plane in the TBAD group, which is equivalent to the LCA pulling more toward the cephalic direction (*p* < 0.05). On the other hand, for the LSA, differences were observed between the TBAD and CS groups in both the (para-)sagittal and (para-)coronal planes. In the (para-)sagittal plane, the take-off angle was less acute in the TBAD group, while in the (para-)coronal plane, the take-off angle was more obtuse than in the CS group (*p* < 0.05). In other words, the origin of the LSA pulled more toward the cephalic direction and backwards in the TBAD group (Table 2).

Furthermore, the distance between the proximal edges of the ostium of each supra-aortic branch was measured. Here, we found increased inter-vessel distance between all three supra-aortic branches in the TBAD group, as opposed to the CS group (Table 2).

### 3.3. Compression and Torsion Index

**Figure 4:**
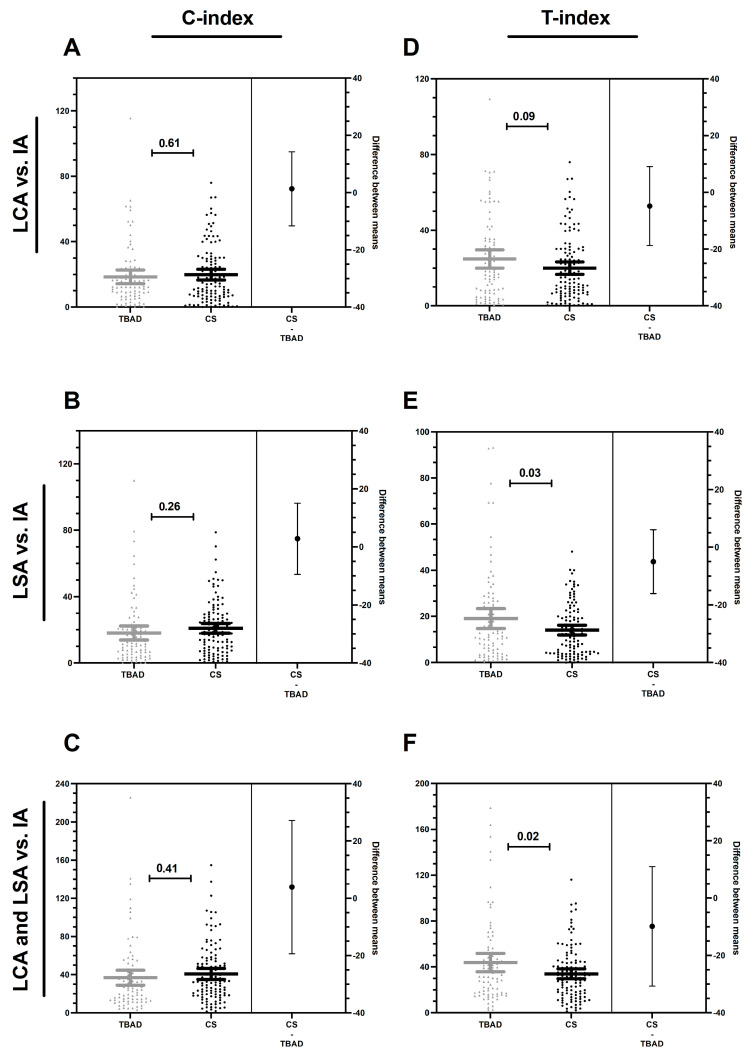
Compression (C-index) and torsion index (T-index). The C-index and T-index were calculated from the para-sagittal and para-coronary take-off angles of the supra-aortic branches to quantify the different spatial configuration. C-index 1 (**A**) describes the deviation of the para-sagittal take-off angle of the left carotid artery (LCA) vs. the innominate artery (IA), C-index 2 (**B**) describes the deviation of the para-sagittal take-off angle of the left subclavian artery (LSA) vs. the IA, and C-index total (**C**) quantifies the deviation of the para-sagittal take-off angle of the LCA and LSA vs. the IA. T-index 1 (**D**) describes the deviation of the para-coronal take-off angle of the LCA vs. the IA, T-index 2 (**E**) describes the deviation of the para-coronal take-off angle of the LSA vs. the IA, T-index total (**F**) quantifies the deviation of the para-coronal take-off angle of the LCA and LSA vs. the IA. P-values indicated in the graph. Significant *p*-values are bold italics. The data is presented as mean with 95% confidence interval (CI). TBAD: type B aortic dissection, CS: carotid stenosis.

To assess inter-group differences in the supra-aortic branch take-off angles with respect to the aortic arch configuration, we formed a C-index and T-index. These indexes allow for the evaluation of branch deviation of the LCA and LSA as opposed to the IA and, therefore, the compression or torsion of the aortic arch, which may result from a deviating outflow configuration of the individual supra-aortic branches relative to each other. Interestingly, for all C-indexes, there was no difference when comparing the TBAD and CS groups; this also applied to T-index 1 (Figure 4A–D). In contrast, T-index 2 and the cumulative T-index total (Figure 4E,F) showed a significant increase in the TBAD group (*p* < 0.05), which may suggest a potential influence of deviated or distorted outflow configuration on the supra-aortic branches supplying the left half of the body as opposed to the IA supplying the right half of the body, which, in turn, can predispose patients to the development of TBAD. Furthermore, the results emphasize the possible higher importance of the resultant torsion of the take-off angle configuration of the supra-aortic branches in the coronal plane compared with that of the compression in the sagittal plane.

## 4. Discussion

The results of the present study suggest that the spatial geometric configuration of the aortic arch and, in particular, the supra-aortic branches may predispose patients to TBAD. In this context, the spatial take-off angle configuration of the left-sided supra-aortic branches, namely, the LSA and LCA, may seem to be of high relevance for the formation of an intimo-medial entry tear. In addition to this specific geometric configuration, the distance between the supra-aortic branches may be another significant contributing factor. Taken together, our measurements indicate a configuration in TBAD patients in which the supra-aortic branches pull the aorta more cranially, while at the same time pulling it more dorsally and leading to more torsion. In combination with the gothic arch configuration, this results in a steeper and more tortuous arch geometry; this, in combination with other stressors, such as elevated blood pressure, may lead to high shear stress in the bifurcating area close to the LSA origin, the predominant primary entry tear location in TBAD.

The geometry of the aorta itself has implications for the development and progression of aortic disease. It has been shown that aneurysms of the ascending aorta and the abdominal aorta are affected by vessel geometry and the biomechanical parameters derived from it [11,12]. For TBAD, this relationship has not yet been sufficiently clarified, especially with regard to individual geometry-associated risk predisposition.

During the last decade, researchers have increasingly addressed the question of which morphologic parameters contribute to the development of dissecting aneurysms following an acute event of TBAD. In this regard, van Bogerijen et al. found that dissection-specific parameters, such as size and location of the entry tear, aortic diameter on admission, and perfusion status of the false lumen, at the time of the acute event, significantly affected outcome parameters during follow-up [13]. Data that enable primary prevention are of crucial importance. However, purposeful concepts have been insufficiently elaborated to date. Importantly, the first evidence in the currently available literature suggests that a bovine (or gothic) arch should be considered a potential risk factor for the development of a thoracic aortic aneurysm [14]. Further, a higher incidence of bovine arches in patients requiring surgical treatment for traumatic isthmic aortic transection was reported [15]. Pointing in the same direction, Sun et al. found that the greater angulation and tortuosity of the aortic arch is associated with TBAD [16]. 

We found the torsion index to be significantly increased in TBAD patients compared with non-TBAD patients, indicating a potentially more distorted aortic blood flow through the outflow into the supra-aortic branches. In fact, a recent study found aortic arch tortuosity to be significantly increased in patients with bicuspid aortic valves, who are at higher risk for AAD development [17], which could result from the more turbulent flow in the aortic arch described by others in these patients [18]. Additionally, increased tortuosity is a common feature in patients with Loeys–Dietz syndrome, who have a high prevalence of AAD [19].

In line with the aforementioned findings, the present study is the first to suggest a potential impact of the spatial geometric architecture of supra-aortic branches on developing TBAD. Mechanistically, this finding seems plausible for various reasons. Wall shear stress (WSS) occurring in the region distal to the LSA has been linked to TBAD development and progression and is believed to be directly affected by the geometric configuration of supra-aortic branches [20,21,22]. It is noteworthy that four-dimensional flow MRI studies suggest higher WSS in the descending aorta when compared with the aortic arch, suggesting a higher mechanical stress in the aortic segment where intimo-medial endothelial tears occur most frequently [23]. Another study underlining the role of WSS as the main determinant of TBAD was introduced by Wen et al., who reported that neither the enhancement nor the oscillation of WSS but rather the multidirectional WSS distribution is most relevant when analyzing type III aortic arch configurations in the context of TBAD [24]. 

Although a direct relationship between the configuration of the supra-aortic branches and the WSS was not the subject of the present work, the results should encourage scientists to investigate this relationship in more detail by applying computational fluid dynamics (CFD) and fluid-structure interaction (FSI) simulations in the future.

The spatial configuration of the supra-aortic branches may also interfere with biological processes that contribute to adverse aortic remodeling and subsequently to increased aortic stiffness. Such a concept of mechanobiological coupling seems plausible in the context of TBAD, considering that Phalla O et al. claimed that an angulated gothic aortic arch is associated with increased central aortic stiffness [25]. Interestingly, aortic stiffness measurements have recently been suggested as an impactful metric for predicting aortic dissection and quantifying dissection risk [26]. While stiffness-related remodeling mostly affects the medial aortic layer, recent observations also suggest increased rigidity of the endothelium in response to risk factors as a potential contributing factor to TBAD development [27]. 

Although the results of the present work did not throw light on the mechanistic relationship between aortic arch geometry and increased aortic stiffness, the data encourage further work in this direction. In the long run, gathered data may help identify aortic arch configurations in patients at risk of TBAD, particularly in a patient cohort with established risk factors, such as the male gender between 60 and 70 years of age suffering from arterial hypertension, engaged in tobacco consumption, or with a history of aneurysmal disease and/or aortic valve deficiencies [28]. In fact, such a cohort may benefit from more stringent monitoring, the adjustment of modifiable risk factors, and more aggressive medical treatment before the onset of TBAD in the sense of a primary prevention concept. This process may, in the future, be effectively supported by KI-driven algorithms. To this end, the authors explicitly agree with Hughes, who stated that the current lack of screening makes primary prevention the most effective strategy for reducing the mortality of aortic dissection [29]. 

To the knowledge of the authors, this study, for the first time, suggests the possible influence of the supra-aortic take-off angle configuration as an implication of directional traction of the branches altering aortic arch configuration and, thus, potentially the likelihood of TBAD development, although further mechanistic clarification of this early data remains to be conducted.

Additionally, this study has major limitations. First, we analyzed CAT scans taken after the onset of TBAD, given the lack of available CAT scans in a sufficient number of patients prior to AAD. It is likely that during the initiation of TBAD and subsequent early-stage remodeling, the aortic arch geometry changes significantly, thus affecting the divergent arch configuration observed between the study groups. Furthermore, the etiologies in both study groups were associated with atherosclerosis; however, our patient populations differed in relevant characteristics. This is also a bicentric study, and generalizations of the results should be verified in a larger patient population.

In conclusion, our results suggest an association between several geometric aortic arch parameters and the presence of TBAD. Further functional studies are needed to verify the pathogenetic relevance of our results and their disease-specific causality. Our results could serve as the first basis for the identification of aortic arches at risk and thus contribute to the reduction in the incidence of TBAD in the long run.

## Figures and Tables

**Table 1:** Patients’ demographics and characteristics. Data are presented as absolute frequencies (percentages) or mean ± standard deviation (SD). *p*-values are presented for type B aortic dissection (TBAD) vs. carotid stenosis (CS), applying Student’s *t*-test, Mann–Whitney-U or Chi-square test where applicable. Significant *p*-values are in bold italics.

	TBAD (N = 85)	CS (N = 114)	TBAD vs. CS *p*-Value
Age (years)	61.5 ± 13.1	71 ± 9.3	<0.000
**Age groups (years)**			
<34	3 (3.5)	-	***0.04***
35–49	9 (10.6)	-	***0.000***
50–64	40 (47.1)	27 (23.7)	***0.001***
65–79	26 (30.6)	64 (56.1)	***0.000***
80–95	7 (8.2)	23 (20.2)	***0.02***
**Sex**			
Male	63 (74.1)	82 (71.9)	0.73
Female	22 (25.9)	32 (28.1)	0.73
**Medication**			
Beta-Blockers	61 (71.8)	68 (59.6)	0.07
ACE-inhibitors	36 (42.4)	60 (52.6)	0.15
AT1-blockers	11 (12.9)	30 (26.3)	***0.02***
Calcium-antagonists	48 (56.5)	63 (55.3)	0.86
Diuretics	35 (41.2)	44 (38.6)	0.71
**Comorbidities**			
aHT	66 (77.6)	107 (93.9)	***0.001***
T1DM/T2DM	5 (5.9)	25 (21.9)	***0.002***
CHD	24 (28.2)	38 (33.3)	0.44
History of tobacco use (prior and current)	12 (14.1)	44 (38.6)	***0.000***
**Dimensions**	**N = 66**	**N = 111**	**N = 177**
Height (cm)	177.7 ± 9.5	172.8 ± 7.9	***0.000***
Weight (kg)	85.7 ± 21.7	80.9 ± 12.7	0.28
BMI (classes)	26.9 ± 5.6	27 ± 3.7	0.35
Underweight	3 (4.5)	2 (1.8)	0.43
Normal weight	19 (28.8)	34 (30.6)	0.24
Overweight	29 (43.9)	51 (45.9)	0.16
Obesity I°	10 (15.2)	23 (20.7)	0.11
Obesity II°	3 (4.5)	1 (0.9)	0.19
Obesity III°	2 (3)	-	0.10

TBAD: type B aortic dissection; CS: carotid artery stenosis; aHT: arterial hypertension; T1DM: type 1 diabetes mellitus; T2DM: type 2 diabetes mellitus; CHD: coronary heart disease. BMI: body mass index. Not all data was available for all patients.

**Table 2:** Aortic arch morphology. Data are presented as absolute frequencies with percentages or mean with standard deviation (SD), and 95% confidence interval (CI). Results are presented for patients with type B aortic dissection (TBAD) and carotid stenosis (CS). The take-off angles for the supra-aortic branches are presented in the sagittal (sag.) and coronal (cor.) plane and measured against a horizontal. The aortic arch angulation is measured in the sagittal plane. The inter-vessel distance was measured between the proximal edge of each ostium. *p*-values are presented for TBAD vs. CS, applying Student’s *t*-test or Mann-Whitney-U or Chi-square-test where applicable. Significant *p*-values are in bold italics.

			TBAD					CS			TBAD vs. CS *p*-Value
**Aortic Arch Classification**	**N**	**Absolute Frequency (%)**				**N**	**Absolute Frequency (%)**				
**Type I**	85	15 (17.6)				114	29 (25.4)				0.18
**Type II**	85	13 (15.3)				114	41 (36.0)				***0.001***
**Type III**	85	57 (67.1)				114	44 (38.6)				***0.000***
**Angles (°)**	**N**	**Mean**	**SD**	**Upper limit**	**Lower limit**	**N**	**Mean**	**SD**	**Upper limit**	**Lower limit**	
**IA sag.**	85	80.6	17.14	84.3	76.9	114	83.8	16.24	86.8	80.7	0.12
**IA cor.**	85	96	20.6	100.4	91.6	114	95	20.4	98.8	91.2	0.97
**IA p-sag.**	85	85	19	89.1	80	114	86.9	16.5	90	83.9	0.44
**IA p-cor.**	85	102.8	22.8	107.7	102.3	114	97.4	16.6	100.5	94.3	0.05
**LCA sag.**	85	70.6	14.1	73.7	67.6	114	76	17.1	79.2	72.8	0.13
**LCA cor.**	85	71.3	17.8	75.2	67.5	114	64.34	15.7	67.2	61.4	***0.001***
**LCA p-sag.**	85	71.7	14.8	74.9	68.5	114	68.7	13.5	71.2	66.2	0.12
**LCA p-cor.**	85	83.6	20	88	79.3	114	73	15.1	75.8	70.2	***0.000***
**LSA sag.**	85	74.6	15.7	78	71.2	114	71.1	14.3	73.8	68.5	0.11
**LSA cor.**	85	84.2	14.3	87.3	81.1	114	79.1	12.1	81.3	76.8	***0.007***
**LSA p-sag.**	85	74.1	13.6	77	71.2	114	69	13.6	71.5	66.4	***0.009***
**LSA p-cor.**	85	97.7	17.9	101.6	93.8	114	91.3	12.3	93.6	89	***0.003***
**Arch angulation**	85	64.2	9.7	66.3	62.1	114	74.7	9.4	76.4	72.9	***0.000***
**Distances (cm)**											
**∆ IA–LCA**	85	1.6	0.5	1.7	1.5	114	1.2	0.3	1.2	1.1	***0.000***
**∆ LCA–LSA**	85	1.5	0.5	1.6	1.4	114	1.3	0.4	1.4	1.2	***0.04***
**∆ IA–LSA**	85	3.1	0.8	3.3	2.9	114	2.6	0.5	2.7	2.5	***0.000***

TBAD: type B aortic dissection; CS: carotid artery stenosis; IA: innominate artery; LCA: left carotid artery; LSA: left subclavian artery; sag.: sagittal; cor.: coronal; p-sag.: para-sagittal; p-cor.: para-coronal.

## Data Availability

The data underlying this study is available from the corresponding author upon reasonable request.
